# Dietary Supplementation of Female Rats with Elk Velvet Antler Improves Physical and Neurological Development of Offspring

**DOI:** 10.1155/2012/640680

**Published:** 2012-04-03

**Authors:** Jiongran Chen, Murray R. Woodbury, Jane Alcorn, Ali Honaramooz

**Affiliations:** ^1^Key Laboratory of New Animal Drug Project of Gansu Province, Key Laboratory of Research and Development for Veterinary Pharmaceutics, Ministry of Agriculture, Lanzhou Institute of Animal Science and Veterinary Pharmaceutics, Chinese Academy of Agricultural Sciences, Lanzhou, Gansu 730050, China; ^2^Department of Veterinary Biomedical Sciences, Western College of Veterinary Medicine, University of Saskatchewan, Saskatoon, SK, Canada S7N 5B4; ^3^Department of Large Animal Clinical Sciences, Western College of Veterinary Medicine, University of Saskatchewan, Saskatoon, SK, Canada S7N 5B4; ^4^College of Pharmacy and Nutrition, University of Saskatchewan, Saskatoon, SK, Canada S7N 5C9

## Abstract

Elk velvet antler (EVA) has a traditional use for promotion of general health. However, evidence of EVA effects at different lifestages is generally lacking. This paper investigated the effects of long-term maternal dietary EVA supplementation on physical, reflexological and neurological development of rat offspring. Female Wistar rats were fed standard chow or chow containing 10% EVA for 90 days prior to mating and throughout pregnancy and lactation. In each dietary group, 56 male and 56 female pups were assessed for physical, neuromotor, and reflexologic development postnatally. Among the examined physical developmental parameters, incisor eruption occurred one day earlier in pups nursing dams receiving EVA. Among neuromotor developmental parameters, duration of supported and unsupported standing was longer for pups nursing EVA supplemented dams. Acquisition of neurological reflex parameters (righting reflex, negative geotaxis, cliff avoidance acoustic startle) occurred earlier in pups nursing dams receiving EVA. Longterm maternal EVA supplementation prior to and during pregnancy and lactation accelerated certain physical, reflexologic, and neuromotor developmental milestones and caused no discernible adverse effects on developing offspring. The potential benefits of maternal EVA supplementation on postnatal development warrants further investigation to determine whether EVA can be endorsed for the promotion of maternal and child health.

## 1. Introduction

Velvet antler is a well-known Chinese *materia medica* used clinically as an ancient oriental natural product for thousands of years in the treatment of various diseases and as a tonic [1]. In the 1990s, North American elk farming for the purpose of velvet antler production was a growing industry bolstered by an increasing demand for elk velvet antler (EVA). However, chronic wasting disease, a prion disease, in farmed elk and deer closed many of the traditional markets to North American antler products. A demand for EVA for Traditional Chinese Medicine (TCM) has, nevertheless, remained, and dried antler is the most frequently used animal-derived ingredient in TCM prescriptions. Recently, the North American holistic health and natural product industry is rediscovering antler's beneficial effects, promoting its use as a food supplement and nonpharmaceutical therapeutic agent for both human and veterinary medicine applications.

According to the principles of TCM, velvet antler has a variety of beneficial health effects. It is prescribed by TCM doctors to enhance the sense of well-being and vitality, improve musculoskeletal function, elevate resistance to disease, and modulate the immune system to decrease allergic response. It is believed also to improve blood circulation, modify blood pressure, and promote rapid healing of tissues and bones [2–4].

Elk velvet antler is harvested from live elk in late spring or early summer while still in its growth phase. Antlers are appendages that grow annually from antler generating structures called pedicles located on the frontal skull bone of male elk and deer. The antler in the growing stage is a soft, cartilaginous, blood-filled tissue covered by skin with a velvet-like texture, hence the name velvet antler. Antlers grow very rapidly reaching growth rates of approximately 2 cm/day. Growth and mineralization is complete at approximately 120 days after it begins. Antlers reach peak commercial value at approximately 70 to 80 days of growth; after this time, the economic value and medical usefulness of antler is gradually lost due to progressive mineralization and concomitant loss of biological activity. The soft, blood-filled cartilage tissue in the distal portions of antler will be replaced by mineralized cartilage and bone tissue advancing up from the base within this period [4–6].

In North America, the processed antler is ground and encapsulated in varying milligram amounts and sold over the counter [7, 8]. In the East, the antler is cut into wafer-thin slices and combined with other natural ingredients in prescriptions for various TCM purposes.

 Despite a long history and widespread use, to our knowledge, little data is available about the effect of EVA on physical and neurological development of neonates particularly following maternal dietary supplementation with EVA. One study evaluated short-term pre- and postnatal exposure of rat pups to EVA (from the 18th day *in utero* to 86 days after birth) and reported no changes in normal physical, developmental, or behavioral indices [2]. To add to the limited database of EVA effects during postnatal development, our study was designed to comprehensively evaluate the effects of long-term maternal EVA supplementation on physical, reflexological, and neurological development of offspring and whether gender differences existed in the offspring's response to maternal supplementation.

## 2. Materials and Methods

### 2.1. Animal Diets

The control diet consisted of regular rodent chow (Prolab RMH 3000, PMI Nutrition International, St. Louis, MO, USA) ground into powder using a laboratory mill. A 10% w/w EVA diet [2] was made using 1 : 9 EVA powder : powered regular rat chow and mixed to homogeneity. EVA powder was derived from the freeze-drying method and was supplied by Norelkco Nutraceuticals (Moosomin, SK, Canada). Briefly, the velvet antler was frozen immediately after harvesting, transported to the plant where the button end (about 2 cm) was cut off in order to help control bacteria and the hide (skin) was removed. The rest of the frozen stick was shredded onto stainless steel trays to expose maximum surface area. The shred was quickly (about 12 h) dried by a blast of cold (−1°C), dry air, which results in minimum loss of bioavailable nutrients and no pathogen buildup. The dried shred was then ground in a nitrogen-cooled grinder to a fine powder.

### 2.2. Animals

Sixteen Wistar adult female rats weighing 200 to 250 g and 16 Wistar adult male rats weighing 400 to 450 g were obtained from Charles Rivers (Montreal, PQ, Canada). Male and female rats were randomly assigned to the two treatment groups and housed in plexiglass cages lined with sawdust. Rats were provided with water *ad libitum* and fed regular powdered rat chow (female and male controls) or 10% EVA powdered chow (male and female treatment groups) *ad libitum* for 90 consecutive days prior to mating. All animals were kept under constant temperature at 21°C and 60% humidity and on a 12 h : 12 h light : dark schedule. Female rats were bred to males fed the same chow as the females. Female and male rats were paired for 5 days or until a vaginal mucus plug (as evidence of mating) was confirmed. Females were checked twice daily until pups were born. The day of birth was designated postnatal day 0 (PND 0). During pregnancy and lactation, the female adult rats from either group continued to receive the same diet as before parturition. All females were allowed to deliver naturally and rear their young to weaning.

After parturition, pups were housed with their dam for a lactation period of 21 days (PND 21), after which they were weaned and provided *ad libitum* with the same chow as their mothers for the remainder of the experimental period of 31 days (PND 31). In order to prevent maternal responses from playing a role in litter manipulations, efforts were made to ensure the early living environment contributed by maternal behavior was the same across groups [9–11]. Pups born to dams fed EVA or regular chow were selected for study across all litters in each diet group to ensure equivalent nutritional intake and maternal care for pups. On PND 2, litters were sexed and 224 pups were assigned to one of 14 pup groups (7 control and 7 EVA groups, corresponding to the diet fed to their mothers), each group containing 8 males and 8 females. This work was approved by the University of Saskatchewan's Animal Research Ethics Board and adhered to the Canadian Council on Animal Care guidelines for humane animal use.

### 2.3. Examination of Physical Development

At parturition, litter size, total litter weight, and mean birth weight of the pups were recorded for each dam. Pups were weighed every day from PND 0 to PND 21. Physical development and maturation of the pups within each dietary group was evaluated by observing the dates of onset and completion of pinna unfolding, incisor eruption, eye opening, and growth of anogenital hair [12–19].

### 2.4. Examination of Neuromotor Development

Crawling, head waving, and supported and unsupported standing were examined as indicators of neuromotor development. Mean age of onset and completion of the indicators in each pup group, the number of animals in each group displaying the indicator activity from day of onset to day of completion, and the time spent in these activities were recorded. The times spent in grooming and total relative activity during a fixed test period were also assessed as parameters of spontaneous activity. The equipment and detailed procedures employed in making these observations were previously described [20].

### 2.5. Examination of Neurological Reflexes

The pups were given a battery of tests to evaluate the development of neurological reflexes. This occurred every morning beginning at 9:00 AM from PND 1 to PND 21 and by the same assessor. The pups were tested in the same room as the dam during separation, under soft white light.

The righting reflex was considered positive if the pup turned over to regain a normal posture within 2.5 s of being placed on its back on a hard flat surface. The acoustic startle test was positive if a loud, sharp, buzzer elicited a sudden extension of head and limbs. A positive cliff avoidance test was recorded if the pup turned and crawled away when placed on a bench top with its forepaws extending over the edge. A pup was judged to possess a negative geotaxis capability if, when placed with its head downward on a grid tilted 45° to horizontal, it could rotate a full 180° and climb the grid within a maximum time of 30 s. If the pup could flex its digits to grasp a paper clip used to stroke the forepaw, it was regarded as having achieved bar holding ability or a palmar grasp. A positive vibrissa placing response was recorded if the pup raised its head and extended its forelimbs to grasp the bench surface when lowered by the tail so that the vibrissae touched the bench. The visual placing test was positive if the pup attempted to grasp the bench surface when lowered by the tail to the bench top but without touching it.

Age (days) of onset and completion of the reflex responses described above for each pup were recorded. The number of animals in each group displaying the positive reflex from onset to completion was also recorded daily. Detailed descriptions of these responses have been published elsewhere [14, 17–19, 21].

### 2.6. Statistical Analysis

All data were analyzed using SPSS for windows (version 17.0). Body weight and the time spent standing supported and unsupported were subjected to repeated-measures ANOVA using a general linear model (GLM) considering age and treatment simultaneously. The mean time of onset and completion for acquisition of the activities was subject to a *t*-test for independent samples. The percentage of animals acquiring the physical, neuromotor, and reflex developmental parameters at various postnatal days were subject to a Chi-Square ANOVA using the Crosstab Procedure. The level of significance was set at *P* ≤ 0.05. Relative EVA effects (i.e., the percent difference between EVA and control groups) and gender effects (i.e., the percent difference between male and female groups) were tested using analysis of covariance (ANCOVA).

## 3. Results

### 3.1. Growth and Physical Development

The diet supplemented with 10% EVA was readily consumed by the adult rats. No obvious differences in the weight of food consumed by dams were detected between groups. Pups from EVA supplemented dams showed no apparent teratogenic effects and no postnatal deaths occurred during the experimental period. No significant differences between treatment groups were observed with regard to litter size, litter weight, or individual birth weight. Rate of body weight gain monitored until postnatal day 21 was not different between pups nursing mothers fed regular chow and EVA-supplemented chow (data not shown), and no gender differences were noted in the rate and pattern of body weight gain.

To determine the possible effects of maternal EVA supplementation on physical development, growth indices and achievement of physical development milestones such as pinna unfolding, eye opening, anogenital hair growth, and incisor eruption were assessed. Neither EVA supplementation nor gender influenced the onset and completion of outer ear flap unfolding (postnatal day 3 and 4, resp.), eye opening (postnatal day 13 and 15, resp.), and anogenital hair growth (postnatal day 14 and 18, resp.) (data not shown). However, the onset and completion of incisor eruption occurred earlier in pups nursing dams supplemented with EVA (postnatal day 9 and 11 versus postnatal day 10 and 12, resp.) (*P* < 0.05) with no differences observed between genders within the respective dietary groups.

### 3.2. Neuromotor and Reflexologic Development

The effects of maternal dietary EVA supplementation on the postnatal day of onset and completion of group acquisition of neuromotor skill and neurological reflex are shown in Table 1. The average age of onset of the neuromotor skills for head-waving (2.0 days) and crawling (4.5 days) occurred at the same age for both groups and genders (data not shown). Pups nursing mothers supplemented with EVA demonstrated earlier onset and completion of several measures of reflex and neuromotor development. Although no statistically significant differences were observed between the diet groups in either age of onset or completion of righting reflex, negative geotaxis, cliff avoidance behaviour, and acoustic startle response, pups from EVA-supplemented mothers tended to develop these activities sooner and more quickly than the control pups (Tables 1 and 2). The mean day of complete group acquisition for both supported and unsupported standing was earlier (*P* ≤ 0.05) in the EVA supplemented group. Few gender differences were noted except where the mean day of onset for unsupported standing in EVA males was earlier than that of the control group, but this difference was not seen with EVA females (Table 1).

When acquisition of reflex and neuromotor skills was assessed on the basis of the average total percentage of pups acquiring the specific skill on a particular postnatal day of development (Table 2), a significantly greater number of pups nursing EVA-supplemented dams showed earlier acquisition of the measured indices. Additionally, pups from EVA-supplemented dams demonstrated significantly greater ability to stand supported or unsupported, measured by time spent in the activity, than pups from control diet dams (Table 3). However, no significant difference between the genders within either group was noted.

No significant differences between groups were observed in the amount of time spent in grooming during a six-minute observation period or in the onset and completion of bar holding ability, vibrissa, and visual placing, and the proportion of pups showing these reflex activities over time was not different (data not shown).

## 4. Discussion

The primary aim of our study was to investigate the effects of long-term maternal dietary EVA supplementation in the diet of female rats prior to mating, and for the duration of pregnancy and lactation, on the physical, reflex, and neuromotor development of the progeny. We employed a battery of behavioral tests that reflect the developmental maturation of the central nervous system [22] and assessed various parameters of physical development. We used long-term maternal and paternal supplementation with EVA as several studies identify the importance of diet on fetal and postnatal development through diet-mediated epigenetic regulation [23, 24]. Furthermore, we assessed potential gender differences as previous studies have noted differences according to gender in the *in utero* and/or postnatal development of progeny from dams undergoing experimental dietary or drug interventions [25–30].

Assessment of EVA effects at this lifestage is relevant as EVA contains a number of bioactive substances including polyamines, chondroitin sulfate, minerals (calcium, phosphorous, magnesium, iron, and potassium), glucosamine, glycosaminoglycans, and hyaluronic acid as well as all 24 amino acids [31, 32]. Furthermore, growing velvet antler contains neutrophin-3 [33] and a number of other growth factors and proto-oncogene mRNA [34]. Cultured antler cells respond to IGF-1 and IGF-2 [35] and display receptors for estradiol and IGF-1 [36]. Deer antler has been shown to contain biologically active molecules including antinarcotic factor(s) [37], growth promoting polypeptides [38], diaphorase activity [39], and epidermal growth factor [40].

 We observed no discernable adverse effects of long-term maternal EVA supplementation on developmental indices of rat offspring. Our results are consistent with Hemmings and Song [2] who reported that EVA supplemented chow had no long-term effect on the offspring of Fischer rats fed EVA chow from the 18th day of gestation to 88 days after birth. This particular study concentrated on the effect of maternal EVA supplementation from the telophase of gestation throughout the lactation period [2]. Our study suggests that maternal supplementation prior to and throughout pregnancy causes no teratogenic or adverse outcomes on postnatal physical and neurological developmental parameters of offspring.

 In our study, we used typical physical and neurological development parameters [41–44] to assess the effects of long-term maternal EVA supplementation on postnatal development. The physical maturation parameters, pinna unfolding, eye opening, and anogenital hair are developmentally and hormonally controlled [45], but were not significantly affected in either gender by the dam's consumption of EVA. On the other hand, the onset and completion of incisor eruption occurred one day earlier in offspring from EVA-fed dams. Incisor eruption is influenced by epidermal growth factor and transforming growth factor, both of which are found in EVA [46, 47]. Our data suggests that maternal EVA supplementation may modulate the factors leading to incisor eruption in offspring.

In the rat, the first 2 weeks after birth constitute a period of rapid brain growth, a critical phase in neurobehavioral maturation, which corresponds to the last months of human fetal brain growth [48]. Among the battery of tests used in the examination of neurological reflex development, righting reflex, negative geotaxis, cliff avoidance, and acoustic startle were positively influenced by EVA supplementation. Furthermore, the number of pups acquiring these skills in the EVA group showed significant improvement through the observation period compared to those in the control group. Although the earlier developing exploratory activities such as crawling, head waving [49], and grooming were not affected by EVA, pups of EVA-supplemented dams were significantly younger in completing more advanced skills and exhibiting more purposeful movements. For example, the righting reflex is thought to measure the development of dynamic postural adjustments that require the integrity of muscular and motor function and adequate acquisition of symmetrical coordination between left and right sides of the body [50]. Both male and female pups suckling dam receiving EVA diet showed earlier onset and completion of this reflex as compared with control pups. This result indicates that EVA maternal supplementation benefits the development of dynamic postural adjustments and acquisition of symmetrical coordination of the body and has a positive effect on the development of these skills. It has been reported that there is a gender difference in neuronal maturation during brain development [51], but we did not observe a gender difference in any of the tests of neurological reflex development. Furthermore, our data revealed no difference between control and EVA-supplemented pups in the onset and completion of a battery of reflexes that assess maturation of sensory motor cortex and cerebellar and vestibular systems (namely, bar holding ability, vibrissa placing, and visual placing) [52, 53].

## 5. Conclusions

In summary, our study suggests that long-term maternal dietary consumption of EVA has beneficial effects on physical and neurological development of offspring rats. The text on Traditional Chinese Medicine, Pen Ts'ao Kang Mu, written in the 16th century in China, considers EVA a universal tonic and an important medicament for sexual virility. This text does not identify the bioactive component(s) of EVA contributing to these health benefits. Similarly, the bioactive constituents resulting in earlier development of physical, reflexological, and neuromotor skills in offspring of long-term EVA supplementation of dams are not known but EVA polypeptides have been associated with growth promoting activities [38]. Whether these contribute to the acceleration of various indices of physical and neurological development is not known but further research is warranted before the use of EVA as a general tonic for humans during period of pregnancy, parturition, and lactation can be promoted.

## Figures and Tables

**Table 1:** Effects of maternal dietary EVA supplementation on the onset and completion of neuromotor skill and neurological reflex acquisition in rat pups.

Signs^2^	Day of onset^1^				Day of completion^1^			
	Male		Female		Male		Female	
	Control	EVA	Control	EVA	Control	EVA	Control	EVA
Righting reflex	2.0 (0.01)	1.0 (0.05)	2.0 (0.02)	1.1 (0.07)	4.9 (0.25)	3.1 (0.12)	4.9 (0.26)	3.1 (0.12)
Negative geotaxis	4.1 (0.18)	4.0 (0.20)	4.0 (0.20)	4.0 (0.20)	6.9 (0.27)	6.8 (0.28)	6.5 (0.30)	6.5 (0.30)
Cliff avoidance	6.6 (0.30)	6.3 (0.26)	6.9 (0.20)	6.9 (0.33)	8.0 (0.35)	6.6 (0.28)	7.9 (0.32)	7.9 (0.35)
Acoustic startle	11.0 (0.45)	9.4 (0.40)	11.0 (0.45)	9.4 (0.40)	12.0 (0.50)	11.0 (0.45)	12.0 (0.50)	11.6 (0.51)
Supported standing	8.0 (0.34)	7.6 (0.33)	8.0 (0.34)	7.6 (0.33)	11.2 (0.65)	10.0 (0.00)*	10.9 (0.48)	9.1 (0.34)*
Unsupported standing	15.9 (0.66)	14.9 (0.65)*	15.8 (0.56)	15.0 (0.70)	17.9 (0.66)	15.8 (0.30)*	17.9 (0.66)	16.1 (0.40)*

^1^Data are expressed as mean ± standard error of man (SEM).

^2^The acquisition of various reflexes (yes/no) was assessed at specific postnatal ages, for example, Righting Reflex at PND 1–5, Negative Geotaxis at PND 4–6, Cliff Avoidance at PND 6–8, Acoustic Startle at PND 9–12, Supported standing at PND 7–11, and Unsupported standing at PND 15–18.

*Significant (*P* ≤ 0.05) differences between control and EVA groups (within gender) in the onset or completion of physical and neurological signs of development.

**Table 2:** Percentage of rat pups possessing an acquired neuromotor and neurological reflex on specific postnatal days.

		% of pups					
Signs		Male		Female		Diet effect (*P* value)	Gender effect (*P* value)
		Control	EVA	Control	EVA		
Righting reflex	PND 1	0	10.7	0	5.4	0.002	NS
	PND 2	28.8	80.4	39.3	71.4	<0.001	NS
	PND 3	53.6	100	76.6	100	<0.001	NS
	PND 4	96.4	100	94.6	100	0.024	NS

Negative geotaxis	PND 4	23	25	35	38	0.019	NS
	PND 5	56	54	70.2	71	0.035	NS
	PND 6	82	85	100	100	0.004	NS

Cliff avoidance	PND 6	10.7	30.3	14.3	26.7	0.003	NS
	PND 7	50	70	55	73.2	0.004	NS

Acoustic startle	PND 9	0	16.1	0	19.6	<0.001	NS
	PND 10	5.4	42.9	14.3	53.6	<0.001	NS
	PND 11	53.6	100	41	93	<0.001	NS

Supported standing	PND 7	0	26.8	0	26.8	<0.001	NS
	PND 8	35.7	7.8	36	73.2	<0.001	NS
	PND 9	62.5	100	59	100	<0.001	NS
	PND 10	73.2	100	89.3	100	<0.001	0.039

Unsupported standing	PND 15	0	44.6	0	21.4	<0.001	0.019
	PND 16	33.9	100	39.2	75	<0.001	NS
	PND 17	74.9	100	66.1	100	<0.001	NS
	PND 18	100	100	89.3	100	0.013	0.013

NS: not significant.

PND: postnatal day, recorded as the first PND where a significant difference (*P* ≤ 0.05) was noted.

**Table 3:** Effect of maternal dietary supplementation of elk velvet antler on neuromotor coordination (standing) of rat pups.

Dietary group	Time spent standing^†^							
	Supported (PND7–11)					Unsupported (PND16–18)		
	PND 7	PND 8	PND 9	PND 10	PND 11	PND 16	PND 17	PND18
Male								
Control	0.0 (0.00)	4.4 (0.21)	7.4 (0.31)	7.2 (0.26)	10.2 (0.65)	6.5 (0.33)	9.6 (0.46)	12.3 (0.55)
EVA	3.4 (0.11)**	4.2 (0.25)	8.1 (0.33)*	10 (0.45)*	11.1 (0.67)	9.8 (0.44)*	13.7 (0.61)*	18.7 (0.77)*

Female								
Control	0.0 (0.00)	4.3 (0.23)	6.8 (0.27)	7.3 (0.30)	10.5 (0.53)	6.8 (0.28)	8.9 (0.36)	13.6 (0.54)
EVA	3.2 (0.12)**	4.5 (0.24)	8.4 (0.40)*	9.8 (0.40)*	11.3 (0.56)	10.2 (0.57)*	13.3 (0.58)*	19.1 (0.70)*

^†^Time in seconds during a six-minute observation period. Each value represents the mean of 56 pups ± standard error of mean (SEM).

The level of significance was set at *P ≤ 0.05 or **P < 0.001.
